# The potential for immunoglobulins and host defense peptides (HDPs) to reduce the use of antibiotics in animal production

**DOI:** 10.1186/s13567-018-0558-2

**Published:** 2018-07-31

**Authors:** Albert van Dijk, Chris J. Hedegaard, Henk P. Haagsman, Peter M. H. Heegaard

**Affiliations:** 1 Division Molecular Host Defence, Department of Infectious Diseases and Immunology, Faculty of Veterinary Medicine, Utrecht University, Utrecht, The Netherlands; 2 Innate Immunology Group, National Veterinary Institute, Technical University of Denmark, Kongens Lyngby, Denmark

## Abstract

Innate defense mechanisms are aimed at quickly containing and removing infectious microorganisms and involve local stromal and immune cell activation, neutrophil recruitment and activation and the induction of host defense peptides (defensins and cathelicidins), acute phase proteins and complement activation. As an alternative to antibiotics, innate immune mechanisms are highly relevant as they offer rapid general ways to, at least partially, protect against infections and enable the build-up of a sufficient adaptive immune response. This review describes two classes of promising alternatives to antibiotics based on components of the innate host defense. First we describe immunoglobulins applied to mimic the way in which they work in the newborn as locally acting broadly active defense molecules enforcing innate immunity barriers. Secondly, the potential of host defense peptides with different modes of action, used directly, induced in situ or used as vaccine adjuvants is described.

## Introduction

Resistance of microbes to antimicrobial agents is a global threat. An increasing number of pathogenic bacteria has been shown to readily develop resistance against antibiotics (antimicrobial resistance, AMR) of different structural classes. The continuous selective pressure of antibiotic residues in the environment has led to the generation of multi-resistant superbugs, some of which are resistant against every antibiotic known to mankind. In addition, vast amounts of antibiotics related to those used in human medicine are still used in animal husbandry to prevent disease outbreaks, while elsewhere in the world antibiotics are used as growth promotors. For example, in Denmark two-thirds of the total prescribed antibiotics are used in animal production [1]. Similar figures apply throughout the EU [2, 3]. The practice of factory farming in which production animals are kept in high densities and numbers facilitates the generation of AMR reservoirs. Currently, it is not possible to determine the contribution that the use of antibiotics in agriculture is making to the emerging nosocomial AMR. However, there is consensus that minimizing the use of antimicrobials in agriculture is essential to safeguard antimicrobials for human medicine and that alternative strategies are needed to reduce the use of antibiotics in animal husbandry. In particular infectious diseases with a mucosal infection component dominate the veterinary antibiotics demand and alternative strategies to handle such diseases can thus be expected to have a major impact on the total antibiotics usage in animal production and will be instrumental in achieving a significant decrease in the total usage (i.e. animal and human combined) of antibiotics in regions with large intensive animal production sectors.

**Figure 1:**
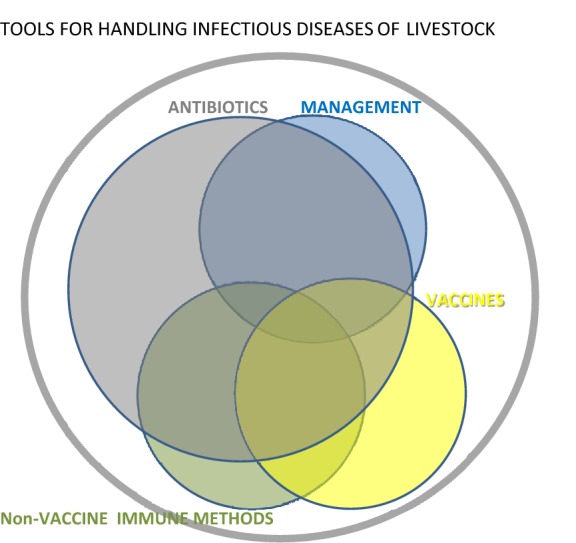
**The outer circle represents all infectious diseases in livestock.** A large proportion of these (namely bacterial infections) can be controlled by antibiotics (grey circle). Some of those can also be controlled by alternative methods such as management measures (blue circle) and/or vaccination (yellow circle). Both of these methods can also be used to control a number of non-bacterial infections not targeted by antibiotics. A significant number of bacterial infectious diseases still remain controllable by antibiotics only, however. We suggest in this review that many of these may be controlled by non-vaccine immune methods, which, given adequate efficiency and low cost may in addition be applicable to some of the infectious diseases that can be handled by management and/or vaccination. As indicated a need for antibiotics will persist. Anyhow, presently available alternative methods can drastically reduce their total consumption and their frequency of use.

As illustrated schematically in Figure 1, bacterial infections will all be treatable with antibiotics. In the animal production sector some will also be treatable or preventable by alternative antibacterial methods such as management measures (e.g. regarding hygiene, animal density, controlling environment and feed etc.) and vaccination [4]. However, a certain proportion will not be treatable by these alternative methods and for those, innate immunology based methods described in this review may be used as alternatives to antibiotics. An important group of such “difficult” infections are infections at mucosal surfaces to which efficient memory immune response are notoriously difficult to raise by vaccination. Importantly, as indicated in Figure 1, a need for antibiotics will remain; however, the implementation of alternative methods will drastically reduce the consumption and frequency of use of antibiotics in animal production, reducing a potential major driver of general AMR development. It should be borne in mind however that, unless specific legislation is imposed, the implementation of any alternative method in the animal production sector depends heavily upon efficiency, ease of use and low cost.

## Innate host defense mechanisms

The generalized host reactions towards infection, aimed at quickly containing and removing the infectious microorganism are collectively known as the innate host defense. The innate immune system is an evolutionary ancient part of host defense. It is present in all organisms; it has a limited repertoire of defense molecules, and a broad specificity. This defense is accomplished by a highly coordinated sequence of events profoundly changing the population of cellular and soluble factors in the affected tissue leading to restored tissue homeostasis, terminating the acute phase of the response and activating adaptive immune responses. Innate defense mechanisms include activation of local stromal and immune cells, the induction of cytokine and chemokine messengers and the resulting attraction and activation of neutrophils/heterophils, macrophages and natural killer (NK) cells, the induction of effector molecules such as enzymes, collectins, acute phase proteins and host defense peptides, and, finally, the activation of the complement system. In addition, we define in this review maternal immunoglobulins acquired by offspring (passive immunity) as temporary innate host defense factors.

**Figure 2:**
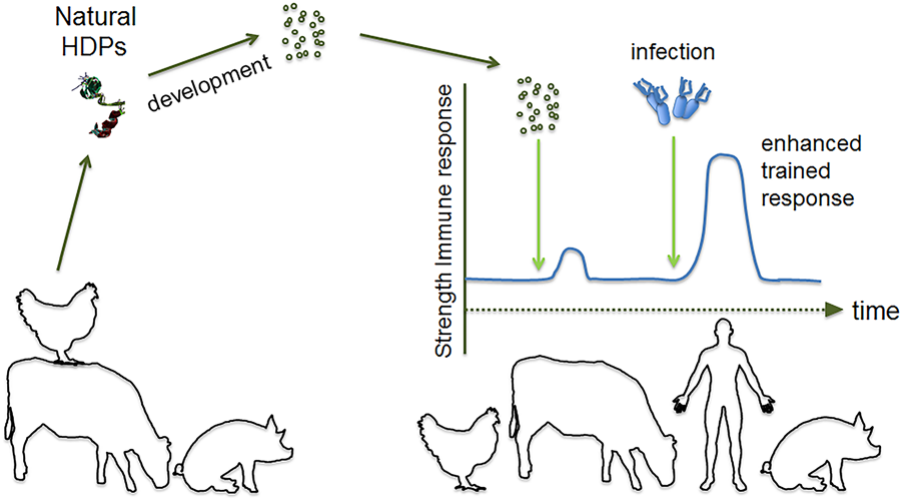
**Trained innate immunity.** Reprogramming of innate immune responses is possible by epigenetic changes induced by compounds like β-glucan. Host defense peptides (HDPs) may induce innate immune memory of monocytes and macrophages in a similar way and increase the threshold above which infection occurs [10]. Trained immunity holds promise as a new approach to decrease the need for antibiotics.

With recent discoveries of adaptive and memory properties of the innate immune system—so-called “trained innate immunity” [5]—the distinction between the innate and adaptive immune systems has become less well-defined. This presents new opportunities for shaping innate immunity and expands the potential of innate immunity based strategies. Trained immunity effects are established quickly (within days) and last for extended periods (months) and manifest themselves as a reprogramming of innate immune responses [6]. Examples include monocytes and macrophages treated with β-glucan or BCG (Bacillus Calmette–Guérin) vaccines becoming hyper-responsive with an increased reactivity towards various, unrelated immune triggers [7]. On the other hand, exposure of monocytes to vitamin A renders them less responsive to microbial ligand stimulation [8]. Dendritic cells, neutrophils, NK cells and other classical innate immune cell types can be affected in similar ways by other types of pathogen associated molecular patterns (PAMPs) of bacteria and other microorganisms [5]. These effects can be observed after resolution of an infection as an altered reaction to a subsequent, unrelated infection [5] and they presumably also are the reason for the “off-target”—effects observed as a side effect of a number of vaccines [6]. Some of these effects are epigenetically based [9] working by modifying histone accessibility and typically affect signal transduction pathways and/or transcription factors, adaptors etc. [10]. Such mechanisms represent unexplored opportunities to “strengthen” immunity which is a desired goal of immune-based intervention with potential to decrease the need for antibiotics; however, before large scale applications in animal farming can be envisaged more needs to be known about basic mechanisms and especially on how specific, desired training effects can be achieved without leading to unwanted effects on innate immune reactivity in general (Figure 2).

Well described soluble components with important functions in the innate immune system include cytokines and chemokines as well as the host defense peptides that are described in detail below and which are remarkable in having both direct antibacterial effects as well as immunomodulatory effects on the host immune system. Cytokines and chemokines can be considered immune system “hormones” with very powerful effects both in the vicinity of the producer cell (auto- and paracrine action) and systemically (endocrine action) [11]. Some of them have highly specific effects such as the chemokines which act as chemo-attractants for neutrophil granulocytes while others have a surprisingly wide range of effects depending on the type of cell binding the cytokine (pleiotropic cytokines) [11]. This latter characteristic together with their endocrine actions (systemic effects) makes the use of certain cytokines as drugs challenging [12]. Even so, some cytokines show early promise (such as IL-22 [13, 14]) and some have been tested successfully for controlling infection in production animals [15]. Thus, bovine G-CSF (granulocyte colony stimulating factor) was reported to have a significantly reducing effect on the number of cattle with clinical mastitis as well as on the absolute neutrophil counts in a herd investigation involving 211 periparturient Holstein cows and heifers given two doses of PEGylated (polyethylene glycol-attached) bovine G-CSF subcutaneously at day-7 and 1 after parturition [16]. One additional major factor preventing the further development and use of cytokines for control of infections in animal production is that it will be hard to manufacture these compounds at an efficiency/price ratio comparable to antibiotics at the doses needed and furthermore that injection-based drugs will generally be less acceptable to the farmers.

This review describes the anti-bacterial mechanisms and possibilities of use as alternatives to antibiotics of two types of molecules representing extremes of the innate immune system and two very different strategies. These alternatives comprise natural immunoglobulin pools obtainable in large amounts from inexpensive sources and intended to be used for oral administration (feed supplement) and host defense peptides, either induced in situ or administered. For a review on immunomodulatory phytochemicals the reader is referred to the article of Lillehoj et al. in this issue.

## Immunoglobulins

### Immunoglobulins as innate host defense molecules

**Figure 3:**
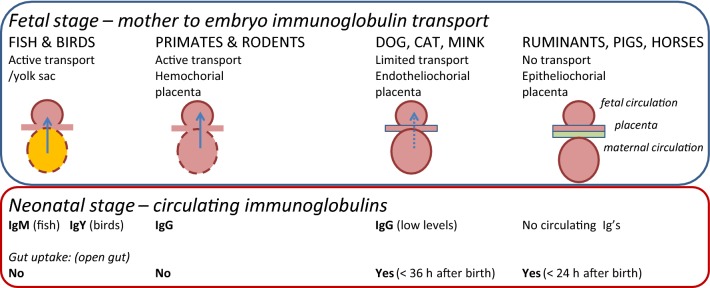
**Transfer of maternal immunoglobulin to offspring is controlled by the interface between the maternal circulation and the placenta (or yolk sac in fish and birds).** Species having an epitheliochorial interface are born without immunoglobulin in the circulation as no transfer takes place during gestation (ruminants, pigs, horses). These species are dependent on uptake of immunoglobulin from the colostrum during the first 24 h after birth and, consequently, their intestine allows immunoglobulin passage in this period, where after it closes. In species with an endotheliochorial interface, neonates have obtained a low circulatory level of immunoglobulin during gestation however are also able to take up immunoglobulins from the gut after being born and up to a week after with the majority of the uptake happening during the first 24–36 h after birth. In primates and rodents, the hemochorial placenta interface allows the neonate to be born with circulating immunoglobulins and there is therefore no perinatal uptake through the gut of maternal immunoglobulin.

Conceptually, pre-existing immunoglobulins (antibodies), which are the effector molecules of humoral immunity, can be conceived as part of the innate immune defense as they reinforce the barriers against infection put up for immediate and general protection by the innate immune system per se. In the neonate, the innate selection of antibodies acquired during gestation through the placental blood supply or post-delivery by ingestion of colostrum and milk is also a part of the innate defense system at the mucosal surfaces of the digestive tract as well as in the circulation. The total pool of immunoglobulins, present or readily produced by primed plasma cells at mucosal surfaces and in the circulation, shares the property of broad reactivity, across a wide variety of microbial pathogens with other innate host defense molecules and systems. In the adult animal, the total immunoglobulin pool is shaped by the cumulative pathogen exposure experienced by the host during its lifetime. In the newborn, immunoglobulins are supplied directly by the mother. This happens in the fetal stage by transplacental transfer supplemented by oral intake of colostrum and milk after birth in animal species having a hemochorial placenta, including primates (Figure 3). In animal species with an epitheliochorial placenta (such as pigs and ruminants) transplacental transfer does not take place and the newborn animal is therefore born without circulating immunoglobulins which must be supplied by the colostrum and milk by lactation (lactogenic immunity) [17]. This principle is used throughout the animal kingdom including birds and fish in which passive transfer of immunity takes place in ovo, dating back at least 450 million years in the evolution [18]. In all cases the newborn is provided with the polyclonal and polyspecific maternal immunoglobulin pool, representing the maternal antibody repertoire induced against the whole spectrum of pathogens experienced by the maternal host throughout her life. For transplacentally supplied immunoglobulins this pool is simply an aliquot of the circulating pool of immunoglobulins in the maternal blood, while in mammals depending on colostrum and milk immunoglobulins the origin depends on the immunoglobulin type. Thus, secretory IgA (sIgA) which is the dominating milk immunoglobulin in primates is mainly produced by local plasma cells in the lymphoid mucosal tissue of the mammary gland. These sIgA producing plasma cells are part of the so-called gut-associated lymphoid tissue (GALT) that also extends to the gut and therefore reflects the antigenic specificities of antibodies induced in the gut. In animal species in which the dominating milk immunoglobulin is IgG (e.g. pigs and cattle), milk immunoglobulins are derived from circulating plasma cells that feed IgG into the mammary gland via an active Fc-receptor mediated transcytosis process that favors specific immunoglobulin classes and subclasses over others, such as IgG1 in bovine [17].

An adequate level of circulatory immunoglobulins has been shown to be of the utmost importance for the disease-free survival of the newborn [19, 20].

In man, the main immunoglobulin type in colostrum and milk is sIgA which is specialized to function at mucosal surfaces such as in the intestinal tract and is not absorbed by the gut. Conversely, in animals born without circulating immunoglobulins IgG is the predominating immunoglobulin type in colostrum and mother’s milk and it is transferred by Fc-receptor mediated active transport from the gut to the circulation in the newborn only within the first 24 h after birth, where after the gut does not allow anymore immunoglobulin absorption. This ensures a very rapid (perinatal) establishment of adequate circulatory levels of immunoglobulins in the newborn providing innate protection against a broad spectrum of infections. Thus maternally derived immunoglobulins protect the offspring until the immune system of the newborn has matured to a state where it can itself respond with adequate adaptive immune responses and build up an immunological memory of its own. In mammals, maternal antibodies have been shown to persist for 2–5 weeks in the offspring (with some variation between species (see Table 1 in [18]).

Immunoglobulins counteract infectious disease by a range of mechanisms including preventing the adhesion and/or entry into host cells of bacteria and viruses, binding to and neutralizing extracellular toxins, enchaining growth of bacteria, accelerating their clearance as shown for IgA [21], opsonizing bacteria i.e. tagging them for destruction by the complement system, and promoting of antibody-dependent, cell-mediated, cytotoxicity against bacteria and viruses [22, 23]. Immunoglobulins are remarkably stable proteins, being digested slowly in the intestinal tract compared to other proteins, IgA being even more stable than IgG [17] and retaining its antigen-binding activity even when partly degraded. Remarkably, bovine IgG_1_ has been found to be just as proteolytically stable as bovine IgA [24].

### Immunoglobulin strategies for control of infectious disease in production animals

The use of immunoglobulins for passive immunization has a long history in both human subjects and animals [25, 26]. The method is currently most often used to treat and/or prevent the effect of bacterial toxins, rabies virus post-exposure and bites and stings of toxic reptiles and scorpions [18]; however, the method is efficient against a broad range of both bacterial and viral infections [27]. A recent illustrative example is the treatment of Ebola virus infection using recombinant monoclonal antibodies (i.e. ZMapp [28]) and convalescent donor plasma having the ability to protect against disease development in human individuals post virus exposure [29, 30]. Immunoglobulins for human use can be administered either as antiserum, i.e. unpurified, as antibodies purified from human serum pools or as purified protein from cell culture (monoclonal antibody based drugs, a rapidly increasing area of drug development), typically using intravenous or intramuscular routes of administration.

A big number of methods exists for producing synthetic immunoglobulins and immunoglobulin derived molecules, for example by plant based expression, by viral expression, allowing administration in feed and upon infection with the virus, respectively. In this review we will purposefully focus on natural immunoglobulins, i.e. immunoglobulin obtained from biological fluids, notably blood and milk, in order to highlight the importance of these often overlooked sources of broad-spectrum antibodies.

Maternal vaccination has been used to protect piglets, lambs and calves against a variety of infectious diseases. Targeted pathogens include a wide variety of bacteria and also a number of viruses demonstrating the broad applicability of the passive immunization principle in immunologically immature stages such as the suckling stage (see [18]).

Also, as mentioned above, a number of licensed, immunoglobulin based products for passive immunization of animals exists (see Table 1, reproduced with permission [18]). A majority of these products is directed against bacterial toxins or bacteria as such; however, an equine IgG product for protecting horses against West Nile virus and a plasma product for restoring defective immunoglobulin plasma levels in horses are also included. Interestingly, many of these products are produced in another species than the target species, the majority is used parenterally and both purified and unpurified immunoglobulins products are available.

**Table 1:** **Licensed products for passive immunization of ruminants, horses and pigs.**

Product type	Animal	Disease prevention/targeted pathogens	Immunoglobulin type/origin	Administration (oral/parenteral)
*E. coli* specific antibodies	Calves	Scour	Bovine colostrum IgG/IgY	Oral
Antibacterial bovine serum antibodies	Cattle	*Arcanobacterium pyogenes*	Bovine serum	Parenteral
	Calves	*E. coli*		
	Sheep	*Mannheimia haemolytica* *Pasteurella multocida* *Salmonella typhimurium*		
Clostridial antitoxins	Cattle	*Clostridium perfringens*	Equine Ig	Parenteral (sc and iv)
	Calves	C&D		
	Goats	*Clostridium botulinum*		
	Sheep	C&B		
	Swine			
	Horses			
Tetanus antitoxin	Horses	Tetanus	Equine serum	Parenteral
	Cattle			
	Sheep			
	Swine			
	Goats			
Anti-West Nile virus antibodies	Horses	West Nile virus	Equine Ig	Parenteral
Anti-endotoxin antibodies	Horses	Septicaemia	Equine plasma from hyper-immune horses	Parenteral
Antibacterial plasma antibodies	Horses	*Rhodococcus equi E. coli* J-5	Equine plasma from hyper-immune horses	Parenteral
Equine plasma	Horses	Failure of passive transfer	Equine plasma	

Reproduced with permission from Hedegaard et al. [18]

Not included in this table is spray-dried plasma (SDP) which is widely used in some animal production sectors for its growth promoting effects and its ability to alleviate widespread production diseases such as post weaning disease (PWD) in weaner piglets [31, 32]. The working mechanism of SDP is not fully known; however to a large extent it can be presumed to depend on its content of active immunoglobulins (approximately 20% of SDP dry weight is immunoglobulin) inhibiting the binding of pathogens to the intestinal mucosa and epithelium, as directly demonstrated [31]. This was further corroborated by the study by Pierce et al. [33] who demonstrated the growth promoting effect of SDP on early weaned pigs to reside in the IgG fraction, confirming that at least a part of the beneficial effect of SDP is due to its content of IgG and its action against intestinal pathogens.

Around 30% of the antibiotics used in the Danish pig production (which demands 75% of the veterinary use of antibiotics) is used to treat PWD [1]. Other big contributors to the veterinary consumption of antibiotics in Denmark and other regions include other intestinal diseases such as diarrhea in newborn and young calves.

In a series of experiments at National Veterinary Institute at the Technical University of Denmark the idea of targeting enteric infectious diseases such as PWD in pigs by oral immunoglobulin administration as a feed supplement was investigated prioritizing low production costs, ease of use and safety, all of which are crucial for an alternative to antibiotics to become widely accepted and used by farmers. While immunoglobulin can be produced using recombinant expression in seeds which can be used as a feed supplement [34] this does not readily allow to obtain the broad range of specificities needed to protect against a wide range of pathogens at the same time, and the following studies therefore focused on immunoglobulin retrieved from natural sources.

First, to keep production costs low the purification of immunoglobulins from natural, inexpensive, easily accessible and processable sources was investigated; for pigs and cattle slaughterhouse blood is a relevant immunoglobulin source being inexpensive and renewable and, importantly having a high concentration (typically > 10 g/L) of immunoglobulins that must be assumed to have relevant specificities as long as the blood is sourced from the same species as intended for treatment and from the same epidemiological area as the target population. Upon slaughter one pig produces 2–3 L of blood which can be immediately collected and stabilized by addition of citrate to yield pig blood plasma. This is a streamlined, hygienic process put in place in most modern abattoirs and often used for producing the raw material for spray-dried plasma (see above) [35]. For poultry and fish a systematic and hygienic collection of abattoir blood is generally not in place; however, blood is evidently also in these cases a major side stream offering a source of highly concentrated immunoglobulin (IgY for poultry, tetrameric IgM for fish). Another inexpensive, renewable source of immunoglobulins is whey, especially of bovine origin. Whey contains roughly 0.7 g/L immunoglobulin, which can be purified quickly by highly efficient methods that are also applicable to blood plasma (see below). In these investigations, for reasons of cost, it was specifically chosen not to prepare hyperimmune serum or whey by active immunization of donor animals; however, a hyperimmunization approach is also feasible if the preferred antigenic specificity is known and if the cost can be kept sufficiently low. Notably, with this approach avian eggs present themselves as containers of conveniently packaged highly concentrated IgY [36]. On average an egg yolk contains 100–150 mg of IgY amounting to at least 20 g of IgY per year per egg-laying hen [37].

Second, highly efficient methods are needed for purifying optimally active immunoglobulin at relatively low costs from large volumes of highly complex starting materials such as blood plasma and whey. This calls for affinity-based methods in the form of industrial scale formats, such as expanded bed adsorption chromatography as well as combined precipitation technologies such as affinity flocculation using polymeric ligands. Both types of processes employ mixed mode affinity ligands with proven group specific binding of immunoglobulins from a range of animal species [38]. These methods can be used to purify immunoglobulins from cattle, pigs, poultry and fish reaching purities in the 80% range in one step (see e.g. [39]) with the added benefit of significantly reducing the concentration of any extraneous agents that might be present, including viruses (Hedegaard et al. unpublished results).

Third, purified immunoglobulin products need to be formulated for controllable and easy oral dosage and for compatibility with automatic feeding and/or drinking systems. In addition, formulation should ensure optimal shelf life at ambient temperature and optimal resistance against the protein denaturing and fragmenting environment of the gut. Last but not least, immunoglobulin products need to be certifiable concerning absence of adventitious agents, including viruses with relevance for pig production such as porcine circovirus type 2 (PCV2), porcine respiratory and reproductive syndrome virus (PRRSV) and porcine endemic diarrhea virus (PED).

Encouragingly, results indicated that purified porcine IgG (ppIgG) obtained from pooled slaughterhouse plasma and purified by expanded bed adsorption chromatography contained antibody reactivity against relevant porcine bacteria (*E. coli* O138, *E. coli* F4 and *E. coli* F18, as well as *Salmonella enterica* Diarizonae) but not against an irrelevant fish bacterium (*Yersinia ruckeri*) [39]. Additionally, it was observed in an *E. coli* O149 F4 challenge model in weaner piglets that piglets given ppIgG orally (4 g/day for 14 days) cleared the challenge strain faster and also had a lower proportion of enterobacteriaceae in their ileal microbiota upon slaughter at the end of the experiment than the control group that did not receive ppIgG [39]. No disease data are available from this experiment as no disease was seen after challenge and therefore a follow-up study was done in which disease was obtained upon challenge with the same *E. coli* type. In this experiment disease was counteracted by ppIgG (oral with feed, 3.8 g/day for 7 days) resulting in less clinical signs of diarrhea and clearance of the challenge strain just as fast as in piglets with access to feed supplemented with dietary zinc oxide for 10 days after weaning (2500 ppm) [40]. Interestingly, pre-feeding ppIgG for 5 days before challenge and continuing treatment for a total of 15 days did not improve protection compared to treatment for 7 days only, starting 1 day before challenge (at the day of weaning). Also of interest was that, while numbers of fecal hemolytic bacteria were reduced by both zinc and ppIgG treatment compared to the untreated group, non-hemolytic levels remained unchanged [40] suggesting a minimal effect of the IgG treatment on the normal microbiota. This would suggest that natural immunoglobulin pools do not contain appreciable activity directed against normal, homeostatic microbiota components, however this will need further investigations to be fully elucidated.

Preliminary work has shown some promising results using immunoglobulin from different sources and applied to other species. In a pilot experiment in which newborn calves were given IgG purified from bovine whey instead of colostrum for the first 24 h after birth the same titer of anti-rotavirus antibodies in the circulation was attained as in the control group having full access to colostrum. In another experiment, bovine immunoglobulin from whey was used as a supplement to colostrum and thereafter as a daily feed supplement for 28 days, leading to total IgG serum concentrations that were higher for the treated group at the end of the experiment (Larsen, Knudsen and Heegaard, unpublished). This shows that purified bovine IgG is readily taken up by the newborn calf. Also, results from this experiment suggested that at least some protection against disease was achieved by the intestinal presence of ingested IgG during the first month of the calf’s life. Other preliminary results showed an effect on campylobacter colonization in chickens in a *Campylobacter jejuni* challenge model, using oral challenge and orally administered purified avian immunoglobulin (IgY) purified from blood (Barnhoff, Hoorfar and Heegard unpublished). This indicates a possible use of the passive immunization principle to reduce the load of zoonotic bacteria in slaughtered animals (with the potential to improve product safety) for example by feeding immunoglobulin during a relatively short period prior to slaughter. These results support the concept that immunoglobulins with relevant activities can indeed be obtained from either slaughterhouse blood or milk/whey of non-immunized animal populations. The principle should be tested as a treatment or prevention option for other hard-to-treat enteric diseases of unknown or multifactorial infectious origin such as porcine epidemic diarrhea (PED), new neonatal porcine diarrhea (NNPD), and mink diarrhea, each of which have a major negative impact on production economy, and animal welfare and which are currently demanding the use of large amounts of antibiotics and/or spray dried plasma.

## Host defense peptides

**Figure 4:**
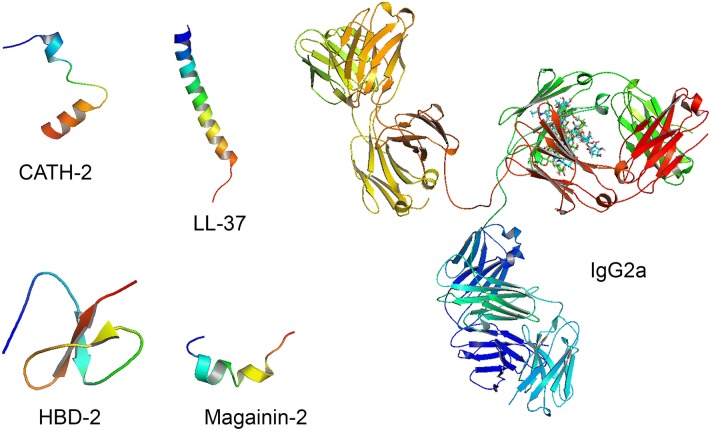
**Three-dimensional representations of structures of chicken cathelicidin-2 (CATH-2), human cathelicidin LL-37, human defensin HBD-2, xenopus magainin-2 and the immunoglobulin IgG2a.** Peptide chains are colored using a color gradient ranging from blue (N-terminus) to red (C-terminus). CATH-2 consists of a double helix separated by a hinge region, LL-37 and magainin-2 adopt a continuous helical structure and HBD-2 consists of an anti-parallel β-sheet structure. The IgG2a structure consists of an Fc fragment (blue/green), two ligand-binding Fab fragments (orange/yellow/green and red/green) and bound polysaccharide ligands NAG-FUC-NAG-BMA-MAN-NAG-GAL-MAN-NAG (blue) and NAG-FUL-NAG-BMA-MAN-NAG-GAL-MAN-NAG (green).

Host defense peptides (HDPs) have an essential role in protecting against microbial challenges due to their presence at host–environment interfaces and broad-spectrum antimicrobial and immunomodulatory activities. Host defense peptides (HDPs) are small peptides that are usually less than 100 amino acid residues long, mostly cationic (+ 2 to + 9 for most peptides) and amphipathic and possess antimicrobial as well as immunomodulatory properties [41]. There are two HDP superfamilies, namely defensins that are β-sheet peptides stabilized by 3 disulfide bridges and can be subdivided into α-, β- and θ-defensins based on the spacing between these cysteine residues, and cathelicidins that are produced as precursor proteins consisting of a signal peptide, cathelin-like domain and a mature bioactive peptide that is proteolytically cleaved off by serine proteases [42]. Cathelicidins can be classified based on the structures they can adopt when interacting with biological membranes, i.e. α-helical peptides (e.g. LL-37), hairpin peptides (e.g. bactenecin), extended peptides enriched in specific amino acids (e.g. indolicidin) [41] (Figure 4).

Although HDPs have been shown to exhibit broad-range antimicrobial activities against Gram-negative, Gram-positive bacteria, fungi, parasites and viruses, including multidrug-resistant strains [41], their capacity to modulate immune cells is increasingly gaining interest. HDPs such as the human cathelicidin LL-37 and human β-defensin-3 (hBD3) have shown to activate monocytes and other immune cells to produce chemokines and cytokines [43–45] and so indirectly stimulate recruitment of immune cells to the sites of infection. Due to their resemblance to chemokines HDPs may also directly attract neutrophils, monocytes, dendritic cells and T cells via C–C chemokine receptor type 2 or 6 (CCR2, CCR6) or *N*-formyl peptide receptor 2 (FPR2) receptors [46–48]. Several HDPs, including LL-37 and chicken cathelicidin-2 (CATH-2), have shown to be anti-inflammatory, capable of neutralizing lipopolysaccharide- and lipoteichoic acid-induced pro-inflammatory cytokine and nitric oxide production [49–52]. Cathelicidins [51, 53] and defensins [54] were also found to enhance DNA-induced activation of macrophages due to enhanced endocytosis of DNA-peptide complexes.

### Regulation of endogenous HDP production

A rich repertoire of HDPs is produced by epithelial cells at mucosal surfaces, skin and immune cells. HDPs are produced by different cells, but the HDP repertoire, cell and tissue distribution is species-specific. For instance, humans produce a single cathelicidin, LL-37, that is ubiquitously expressed and found in epithelial cells, neutrophils, macrophages, dendritic cells, B cells, NK cells and mast cells [55], whereas chicken cathelicidin-2 seems to be exclusively produced by heterophils [42], the avian counterpart of the mammalian neutrophil. Neutrophils and Paneth cells are primary producers of α-defensins, and α-defensins are expressed to a lesser extent by monocytes, lymphocytes and epithelium [55]. β-Defensins are expressed by epithelial cells, monocytes, macrophages, dendritic cells, but have also been found in heterophils and enteroendocrine cells [55–57]. The expression, secretion and activity of HDPs are regulated in various ways, i.e. on the level of developmental transcription, post-translational processing and secretion.

#### Transcriptional control

Some HDPs are constitutively expressed, independent of microbial exposure, such as most gut α-defensins that are transcriptionally regulated via the Wnt pathway, an important signaling pathway [58, 59] and certain β-defensins [60]. Other peptides, including hBD2 (human β-defensin-2), require microbial ligands for full expression [60]. Transcriptional control of cathelicidins is cell-type specific, e.g. microbial induced upregulation in monocytes/macrophages and epithelial cells, while transcription in neutrophils predominantly occurs at the promyelocyte stage [42, 61].

#### Post-translational and secretion control

In humans and rodents, α-defensins are produced as inactive precursor proteins and stored inside granules in neutrophils and specialized enterocytes, so-called Paneth cells that are located in the intestinal crypts [59]. Upon recognition of microbial ligands by pattern recognition receptors at the cell surface granules are released in the crypt lumen. Similarly, cathelicidins and defensins are stored as inactive precursors in a subset of granules in mammalian neutrophils and avian heterophils [42, 62–64]. When recruited to a site of infection, contact with microbial ligands will trigger HDP release and their subsequent activation by serine proteases in the case of cathelicidins [42] and defensins depending on species and tissue amongst others by trypsin, kallikreins or matrix metalloproteinase-7 (MMP-7) [65]. Mammalian enteric β-defensins hBD1 (human β-defensin-1), mBD1 (mouse β-defensin-1) and mBD3 (mouse β-defensin-3) are constitutively produced [60] and released into the gut lumen where they contribute to the chemical barrier formed by the intestinal mucus layer. Other members of the α- and β-defensin families are regulated by microbial ligands [59, 66]. For example, Paneth cell α-defensins are released into the lumen through activation of intracellular nucleotide-binding oligomerization domain-like (NOD) receptors by bacterial muramyl dipeptide (MDP) [65] and neutrophil release of HDPs can be triggered by lipopolysaccharides (LPS) [42], whereas flagellin upregulates hBD2 in skin keratinocytes [67].

#### Developmental control

Abundant cathelicidin expression is present in the skin of human and murine neonates and is downregulated ten- to 100-fold in adults [68]. In chickens, the expression of the cathelicidin, CATH-2, peaks around hatch [57]. The sterile surfaces of skin and mucosae are colonized after birth, and alter during weaning which evokes a shift in the local expression of HDPs. For instance, in mouse intestine, the expression of the mouse cathelicidin-related antimicrobial peptide (CRAMP), decreases, while cryptdin-related sequences (CRS) peptide and α-defensin expression increase with weaning [69]. These changes are important to maintain gut homeostasis as α-defensins have been shown to influence gut microbiota composition [70].

### Antimicrobial strategies involving HDPs

Several HDP-based strategies can be applied that could potentially lead to a reduction of the use of antibiotics in animal husbandry. For example, feed additives can be used to enhance levels of endogenous HDP expression. Alternatively, HDP expression may be enhanced via breed selection or transgene approaches, which will not be discussed in this review. A third option would be to use HDPs as template for the development of novel antimicrobials and immunomodulators. Finally, HDPs may be used as adjuvants for augmenting particular types of immune responses upon vaccination.

#### Induction of endogenous HDP production

Several substances have been shown to upregulate endogenous HDP production and to counteract pathogen-mediated HDP suppression. Therefore, dietary supplementation of food/feed could be used to boost endogenous HDP expression levels and improve the outcome of diseases. Short-chain fatty acids propionate, butyrate, and isobutyrate and the flavanoid flavone are known to regulate colon cell differentiation and increase LL-37 expression in human colonocytes [71, 72]. Polyunsaturated fatty acids (PUFA) induce hBD-1 expression in these cells [73]. Similarly, butyrate analogs can induce defensin and cathelicidin transcription in porcine epithelial cells and macrophages [74]. *M. tuberculosis*-mediated LL-37 suppression in humans could be overcome by treatment with the butyrate analog phenylbutyrate and the vitamin D3 analog 1,25[OH]2D3 separately, while a synergistic action was observed for the combined treatment [75]. In addition, 1,25[OH]2D3 has also been reported to induce β-defensin expression in chicken peripheral blood mononuclear cells (PBMCs) and embryonic intestinal epithelial cells [76]. Oral butyrate treatment of experimental Shigellosis upregulated the production of LL-37 homolog CAP-18 (18-kDa cationic antimicrobial protein) in rabbits and reduced the clinical illness and bacterial load in stools [77]. Similar findings were obtained for phenylbutyrate against Shigellosis [78] and enteropathogenic *E. coli* associated diarrhea [79]. Sulforaphane, a phytochemical produced in cruciferous vegetables, increased hBD-2 transcription in colonocytes [80]. The mode of action of sulforaphane and butyrate analog-induced HDP production is based on inhibition of histone deacetylases (HDACs) leading to chromatin hyperacetylation and increased gene expression [71, 80]. Not surprisingly, the HDAC inhibitor Entinostat increased both LL-37 and hBD1 transcription in a human intestinal cell line. Entinostat-induced LL-37 expression was mediated via the STAT3-HIF1α (signal transducer and activator of transcription 3-hypoxia-inducible factor 1-α) pathway in vitro and impaired in vivo in macrophages obtained from a STAT3 deficient patient [81]. A single dose of live-attenuated oral polio vaccine (OPV) and Bacillus Calmette–Guérin (BCG) vaccine within 48 h of birth increased gut LL-37 production in infants at 6 weeks of age, possibly by OPV stimulation of T cell production of IL-17 (interleukin-17) and IL-22, known regulators of mucosal LL-37 expression [82]. The adenyl cyclase agonist forskolin induced avian β-defensin-9 (AVBD9) expression in chicken crop tissue [83] and LL-37 in undifferentiated human mucosal epithelial cells [84], whereas in butyrate-differentiated mucosal epithelial cells forskolin suppressed defensin and cathelicidin production [85] implicating a role for cyclic AMP in HDP regulation.

Probiotics can also be used to enhance endogenous expression of HDPs. Lactobacillus GG treatment of patients with oesophagitis was found to induce the transcription of several immune-related genes including human α-defensin 1 (HNP1) in duodenal mucosa [86]. Other lactobacillus strains and *E. coli* Nissle 1917 flagellin induced HBD-2 production in Caco-2 cells [87, 88]. In a 3 months-trial involving healthy children receiving *Lactobacillus paracasei*-fermented cow’s milk compared to a placebo group, dietary intake of *L. paracasei*-fermented milk resulted in a net increase in fecal concentrations of LL-37, α-defensins (HNP1–3), and hBD2 that were negatively associated with the occurrence of common infectious diseases, respiratory tract infections and acute gastrointestinal infections [89]. l-Isoleucine induced β-defensin expression that was associated with less tissue damage and lower bacterial loads [90]. Arginine and albumin induced hBD-1 in human colonocytes [73]. Branched amino acids isoleucine, leucine and valine elevated the in vivo transcription of β-defensin-1, -2, -114, and -129 in porcine small intestine [91]. Thus, dietary administration is a possible route to elevate HDP production but care should be taken that a proper balance is maintained to ensure homeostasis.

#### HDP-derived antimicrobials

Due to their broad spectrum of antimicrobial activities HDPs are of interest as a novel class of antimicrobials. Unlike conventional antibiotics which readily induce resistance, in many cases HDPs deploy multiple mechanisms to kill microbes including inhibition of cell division, protein synthesis and DNA replication [64, 92]. A few species, e.g. *Burkholderia* spp. are highly resistant to the direct antimicrobial action of HDPs. However, co-evolution of microbes and HDPs for millennia has not led to ubiquitous resistance against HDPs [93]. Still several pathogens have developed immune evasion strategies to protect against HDPs. The major virulence proteins of enteric pathogens *Vibrio cholera* (cholera toxin) and enterotoxigenic *E. coli* (labile toxin) down-regulate hBD1 and LL-37 production by intestinal epithelium [85]. Similarly, Shigella, a major cause of infant mortality and morbidity in developing countries, is able to down-regulate LL-37 and hBD1 in human rectal epithelium [77]. *Campylobacter jejuni* strains, that are highly susceptible to the chicken CATH-2 peptide that is abundantly present in chicken heterophils, appear to down-regulate CATH-2 expression as part of their immune evasion strategy [94]. Similarly, *Mycobacterium tuberculosis* infection of human macrophages suppresses LL-37 expression and autophagy-related genes at the mRNA and protein level [75].

Interaction between HDPs and the microbial membrane is thought to occur first through electrostatic interaction followed by insertion of hydrophobic groups into the lipid bilayer and transfer in or through the bilayer leading to transient pore formation and binding to RNA, DNA and proteins. Although not entirely impossible, development of systematic microbial resistance to HDPs is greatly hampered by the fact that microbes would need to reorganize their cell membrane composition to avoid peptide binding and membrane permeation. Thus, the same care and prudence involved in conventional antibiotic use should be taken when HDPs are therapeutically used for their direct antimicrobial activity. Several HDP-derived peptides have been tested in preclinical and clinical trials. Synthetic LL-37 has shown efficacy as a topical antibiotic for treatment of “hard-to-heal” venous leg ulcers in phase I/II clinical trials [95]. Analogs of bovine indolicidin (Omiganan/MSI78), frog magainin 2 (Pexiganan/MX-226/MBI-226), porcine protegrin 1 (Iseganan/IB-367) were pursued in phase III clinical trials as a topical antiseptic and treatment of severe acne and rosacea, as topical antibiotic, and as antibiotic against oral mucositis in patients undergoing radiation therapy, and showed a similar efficacy but no advantage to existing therapies [93]. It may be concluded that the development of HDPs as alternative antimicrobials may be more successful for topical rather than systemic use.

#### HDP-derived immunomodulators

A most promising strategy is the use of HDPs as immunomodulators. Under physiological conditions, antimicrobial actions of HDPs may be impaired by the presence of salt, serum and charged molecules (glycosaminoglycans (GAGs), DNA) [96], but despite this, HDPs have been shown to modulate immunity and the function of immune cells [96, 97]. An important feature of HDPs is their capacity to modulate the differentiation of antigen presenting cells, such as dendritic cells and macrophages. Dendritic cells (DCs) are instrumental in coordinating an appropriate T cell response to infections. The environment in which DCs mature greatly influences their phenotype and plasticity. In the presence of LL-37 differentiation of human peripheral blood monocytes to immature DCs resulted in upregulation of antigen presentation markers HLA-DR (human leukocyte antigen-antigen D related) and CD86 (cluster of differentiation 86) [98], whereas LL-37-derived mature DCs exhibited a Th1 (type 1 helper T cells) cytokine profile and stimulated proliferation of IFN-γ (interferon-γ) producing T cells [99]. The chicken cathelicidin CATH-2 was shown to modulate immune responses of chicken mononuclear phagocytes and induced antigen presentation [100]. LL-37 modulation of DC differentiation was G-protein coupled receptor (GPCR) mediated and occurred early in differentiation [99]. The presence of LL-37 during or after differentiation of M2-polarized macrophages (by macrophage colony stimulating factor, M-CSF) skewed macrophage polarization towards a pro-inflammatory phenotype upon LPS stimulation, i.e. CD163^low^, IL-10^low^, IL12p40^high^ [101]. LL-37 did not affect polarization of fully differentiated M1-polarized macrophages (by granulocyte–macrophage colony stimulating factor, GM-CSF), but enhanced GM-CSF-driven macrophage differentiation [101].

Both the α-defensin HNP-1 and the β-defensin hBD-1 promote maturation of monocyte-derived DCs resulting in enhanced expression of maturation marker CD83, antigen presentation markers CD80, CD86, CD40, HLA-DR and scavenger receptor CD91 that also recognize defensins as ligands, suggesting the existence of an autocrine activation loop by which defensins may amplify their own effects [102, 103]. Phagocytosis via integrin Mac-1 of Gram-negative and Gram-positive bacteria by macrophages could be promoted by coating bacteria with LL-37 [104]. HDPs may affect wound healing in several ways, by promoting neovascularization and angiogenesis, stimulating extracellular matrix proteoglycan production, promoting re-epithelization, and managing of the microbial burden through their antimicrobial properties [105–107].

A prime example of the prophylactic use of HDP-derived peptides is avian and fish immunomodulation in ovo. Via this route biological agents are directly injected into the amnion fluid, which is then imbibed by the embryo and distributed throughout the respiratory and gastrointestinal tracts. The in ovo route has several advantages: (a) the peptide concentrations that are used are far below MIC (minimum inhibitory concentration) values, which excludes antibacterial activities and thus the risk of resistance development; (b) a low peptide dose is needed, which is beneficial from a cost of goods perspective; (c) in ovo vaccination of chickens at 18 days of embryonic development (3 days before egg hatch) is commonly used in the poultry industry; (d) this strategy creates a window of opportunity for modulation of the immune system at an early stage. Cuperus et al. demonstrated that in ovo prophylactic treatment with 1 mg/kg body weight of the d-amino acid analog of chicken cathelicidin-2 (DCATH-2) partially protects chickens against a respiratory *E. coli* infection 7 days after hatch [108], resulting in reduced mortality (30%), and reduced morbidity (63%) and respiratory bacterial load (>90% reduction) among surviving birds. Injection of fluorescently labelled DCATH-2 peptide via the in ovo route confirmed that DCATH-2 peptide accumulated, via uptake of amnion fluid, in the lungs and gastrointestinal tract within 24 h post-injection (pi). Similarly, injection of 2.6 ng/kg DCATH-2 into the yolk of 0.2–1.5 h post-fertilized zebrafish embryos delayed infection of a lethal dose of *Salmonella enteritidis* [109]. DCATH-2 treatment of zebrafish embryos in the absence of infection resulted in a marked increase (30%) of phagocytic cells [109]. These findings show that immunomodulation by HDP-derived peptides may cross the species barrier, thus theoretically the same peptide could be used to boost resistance against infectious diseases in multiple species.

Prophylactic application could also be done postnatally. Innate defense regulators (IDRs) are a group of small immunomodulatory peptides with weak or no antibacterial activity that were developed using the bovine cathelicidin bactenecin 2a (RLARIVVIRVAR-NH2) as template. In vivo efficacy has been demonstrated for several IDRs against an invasive *Staphylococcus aureus* and systemic *E. coli* infection. Intraperitoneal treatment of mice with 8 mg/kg IDR-1002 (200 µg/mouse) or 4 mg/kg IDR-HH2 4 h before infection with *Staphylococcus aureus* reduced the bacterial load in peritoneal lavage 24 h pi and was found to be monocyte-dependent and associated with increased leukocyte recruitment and chemokine production [110, 111]. Similar efficacy was observed for IDR-1002 against *E. coli* in this model [110].

#### HDPs as adjuvants for vaccines

HDPs have also gained interest as an adjunct to vaccines for human and veterinary applications. The role of adjuvants in vaccines is crucial as they augment the host immune response against often weakly immunogenic pathogen-derived antigens and are able to selectively bias this response towards a Th1 or Th2 response. Proper adjuvants and adjuvant combinations effectively enhance and modulate the immune response via one or more mechanisms such as by recruitment of immune cells to the administered antigen and enhance antigen presentation by APCs (antigen presenting cells). The multifaceted immunomodulatory properties of some HDPs and HDP-related peptides may be used to “skew” the immune response in the desired direction. This was shown for indolicidin, a short (13 aa) bovine cathelicidin peptide. Immunization of mice by co-administration of OVA (ovalbumin) with indolicidin biased to a type 2 response with increased IgG1 production and number of IL-5 producing cells, whereas co-administration with CpG-DNA and indolicidin at a 1:67 molar ratio augmented both IgG1 and IgG2a production. Addition of polyphosphazene (PP) during immunization with OVA/CpG-DNA/indolicidin further increased IgG2a production by threefold compared to OVA/CpG-DNA/indolicidin alone, suggesting a more balanced immune response [112]. Similarly, indolicidin enhanced the immune response to hen egg lysozyme (HEL) in cattle; re-stimulation of PBMCs obtained 14 days after 2^nd^ immunization showed a higher number of IFN-γ secreting cells after immunization with HEL/CpG/indolicidin compared to HEL/CpG, whereas CpG addition to HEL did not. Immunization with HEL/CpG/indolicidin/PP raised the antigen-specific humoral (total IgG titer in serum) and long-lasting cell-mediated immune responses (number of IFN-gamma secreting cells) [113]. Immunization of mice with pertussis toxin (PT), IDR-HH2 and CpG-DNA (PT/CpG/IDR-HH2) led to a balanced Th1/Th2 response, augmenting toxin-associated IgG1 and IgG2a titers as well as IgA titers, whereas toxin alone (PT) or combined with CpG-DNA (PT/CpG) failed to induce a strong immune response [114]. Immunization with toxin and IDR-HH2 (PT/IDR-HH2) resulted in a Th2 biased response. Oral administration of LL-37-conjugated enhanced green fluorescent protein (EGFP-LL-37) to mice resulted in an enhanced and Th17-skewed T cell dependent antigen-specific antibody response without induction of oral tolerance compared to mice receiving EGFP alone (EGFP), indicating that HDPs may be used as mucosal immune adjuvants [115]. These studies show that HDP-derived peptides can be used as an adjuvant to boost the immune response as well as to skew this response in the desired direction.

#### HDPs as adjuncts in antibiotic therapy

The adjunctive use of HDPs in antibiotic therapy has been examined against experimental tuberculosis, systemic *E. coli* infection and cerebral malaria. *M. tuberculosis* (TB) infected mice were subcutaneously treated 15 days p.i. during 4 weeks, with daily doses of 25 mg/kg of the anti-TB drugs isoniazid and rifampicin and/or a weekly dose of 5 µg/mouse of human neutrophil defensin-1 (HNP-1). Treatment with anti-TB drugs alone reduced the bacterial load by approx. 1 log unit in lungs, liver and spleen, whereas combined therapy with anti-TB drugs and HNP-1 augmented reduction of bacterial loads by eight- to tenfold in lungs and liver and by threefold in spleen [116]. In another study, neutropenic mice were challenged i.p. (intraperitoneal injection) with a lethal dose of *E. coli* and treated with the β-lactam antibiotic cefepime (0.2 mg/kg) or with the HDP magainin 2 (2 mg/mouse) alone raised survival to 20% at 10 days pi compared to 10% in control animals. Combined treatment of challenged mice with cefepime and magainin 2 raised survival to 62.5% [117]. Therapeutic efficacy of HDP-derived peptide IDR-1018 as adjunctive treatment for cerebral malaria was tested in a preclinical model in which mice were infected with *Plasmodium berghei*-infected erythrocytes and on day 4 of infection were daily treated with anti-malarial drugs pyrimethamine and chloroquine up to 11 day pi [118]. Treatment with anti-malarials protected only 41% of the mice, whereas adjunctive therapy with a single i.v. (intravenous injection) dose of IDR-1018 at day 4, 5 and 6 increased survival to 68%. Interestingly, IDR-1018 treatment did not affect parasitemia and its adjunctive protection against late-stage malaria was linked to reduced inflammation. Currently, few immunomodulatory HDP analogs are being pursued in preclinical or clinical trials. In conclusion, HDPs can be used (1) prophylactically in ovo or postnatally, (2) as an adjuvant to vaccines, and (3) therapeutically as adjunct to conventional antibiotics or directly as antimicrobials.

## Conclusions

For antibacterial products based on innate defense molecules to become attractive products as alternatives to antibiotics in animal husbandry it is necessary to match the low cost, efficiency and ease of use of traditional antibiotics. In addition, these products should be broadly applicable, have low adverse effect levels and must be safe, as well as being acceptable to consumers.

Development of host defense peptide-based immunomodulators is a challenge, but with potentially great rewards. The lack of translation of in vitro to in vivo immunomodulatory activities and challenges concerning choice of administration routes makes it difficult and laborious to optimize activities of lead peptides. The costs of large-scale production of synthetic and expressed peptide immunomodulators have decreased and because low doses are needed for immunomodulation costs of goods are, even for veterinary use, no major hurdle. However, the greatest challenge faced is getting immunomodulators approved by regulatory agencies under the current legislation. Approved efficacy and safety tests were developed to evaluate molecules with direct antimicrobial activities; however, these tests are not suitable to evaluate immunomodulators.

In contrast, it will probably be possible to categorize products based on purified natural immunoglobulin pools for oral administration as feed supplements with much lower regulatory hurdles to overcome. Also, production costs can be kept at a level that makes their large-scale use in animal production economically feasible for the producers. However, challenges remain with immunoglobulin based products, including proving efficiency against relevant infections of production animals, obtaining reproducible, stable and consistently active products, optimally formulated for action in the gut and last but not least to ensure the absence of unwanted agents, especially viruses in products produced from blood.

In conclusion, innate host defense mechanisms offer interesting modes of actions for new strategies for counteracting microbial infections and disease in animal husbandry.

Host defense peptides offer several modes of use and—as they have a dual mode of action—may be used with a low risk of inducing AMR. Likewise, immunoglobulins are nature’s own multi-target anti-pathogen effector molecules.

These innate host defense derived molecules provide general and rapid protective measures against infections, delaying establishment, growth and spread of the infection, allowing the adaptive immune system time to develop highly specific and high-affinity cellular and humoral defenses factors taking over protection in time to prevent or significantly slow down disease development. Most importantly, based on anti-bacterial mechanisms tested by the evolution they must be assumed to carry a very low risk of inducing new classes of resistance traits in bacteria and therefore constitute real alternatives to existing antibiotics.

## Abbreviations

AMR: antimicrobial resistance
APCs: antigen presenting cells
AVBD9: avian β-defensin-9
BCG: Bacillus Calmette–Guérin
CAP-18: 18-kDa cationic antimicrobial protein
CATH-2: chicken cathelicidin 2
CCR: chemokine receptor
CD: cluster of differentiation
CRAMP: cathelicidin-related antimicrobial peptide
CRS-peptides: cryptdin-related sequences peptides
DCATH-2: d-amino acid analog of chicken cathelicidin-2
DCs: dendritic cells
EGFP: enhanced green fluorescent protein
FRP: *N*-formyl peptide receptor
GAGs: glycosaminoglycans
G-CSF: granulocyte colony stimulating factor
GM-CSF: granulocyte–macrophage colony stimulating factor
GPCR: G-protein coupled receptor
hBD: human β-defensin
HDACs: histone deacetylases
HDPs: host defense peptides
HEL: hen egg lysozyme
HIF-1-α: hypoxia-inducible factor 1-α
HLA-DR: human leukocyte antigen-antigen D related
HNP1: human α-defensin 1
IDRs: innate defense regulators
IFN-γ: interferon-γ
Ig: immunoglobulin
IL: interleukin
LL-37: human cathelicidin
LPS: lipopolysaccharides
mBD: mouse β-defensin
M-CSF: macrophage colony stimulating factor
MDP: muramyl dipeptide
MIC: minimum inhibitory concentration
MMP-7: matrix metalloproteinase-7
NK cells: natural killer cells
NOD receptor: nucleotide-binding oligomerization domain-like receptor
NNPD: new neonatal porcine diarrea
OPV: oral polio vaccine
OVA: ovalbumin
PAMPS: pathogen associated molecular patterns
PBMCs: peripheral blood mononuclear cells
PCV2: porcine circovirus type 2
PED: porcine endemic diarrhea virus
PEG: polyethylene glycol
PP: polyphosphazene
PRRSV: porcine respiratory and reproductive syndrome virus
PT: pertussis toxin
PUFA: polyunsaturated fatty acids
PWD: postweaning disease
ppIgG: purified porcine IgG
sIgA: secretory IgA
SDP: spray-dried plasma
STAT3: signal transducer and activator of transcription 3
TB: tuberculosis
Th1: type 1 helper T cells
NAG: *n*-acetyl-d-glucosamine
FUC: α-l-fucose
BMA: β-d-mannose
MAN: α-d-mannose
GAL: β-d-galactose
FUL: β-l-fucose
